# Postoperative diagnosis of uterine florid cystic endosalpingiosis: a case report emphasizing diagnostic challenges and multimodal imaging correlation

**DOI:** 10.3389/fmed.2025.1537986

**Published:** 2025-04-15

**Authors:** Yanli Hao, Xiajing Liu, Tingting Wu, Qiuni Liang

**Affiliations:** ^1^Department of Ultrasound, Shenzhen Second People's Hospital, The First Affiliated Hospital of Shenzhen University, Shenzhen, China; ^2^Department of Radiology, Shenzhen Second People's Hospital, The First Affiliated Hospital of Shenzhen University, Shenzhen, China; ^3^Department of Pathology, Shenzhen Second People's Hospital, The First Affiliated Hospital of Shenzhen University, Shenzhen, China; ^4^Department of Gynecology, Shenzhen Second People's Hospital, The First Affiliated Hospital of Shenzhen University, Shenzhen, China

**Keywords:** endosalpingiosis, uterine myometrial cysts, multimodal imaging, contrast-enhanced ultrasound, hormone receptor expression

## Abstract

**Background:**

Endosalpingiosis, a rare benign condition characterized by ectopic fallopian tube-like epithelium, often coexists with endometriosis. This case report presents a unique instance of florid cystic endosalpingiosis confined to the uterine myometrium—marking the first documented case without associated pelvic pathology. Using multimodal imaging and histopathological analysis, we highlight key diagnostic approaches to distinguish this condition from malignant mimics.

**Case presentation:**

A 47-year-old woman with a 2-year history of chronic pelvic pain and recent irregular bleeding underwent surgical exploration after imaging revealed isolated, non-communicating cystic lesions within the myometrium. Histopathological examination identified cyst walls lined with ciliated pseudostratified epithelium, confirmed as benign through immunohistochemistry. A 6-year follow-up showed no evidence of recurrence.

**Conclusion:**

Florid cystic endosalpingiosis should be considered in the differential diagnosis of cystic uterine lesions. Establishing standardized imaging criteria and adopting fertility-preserving management strategies can help avoid unnecessary radical interventions, optimizing outcomes for premenopausal women.

## Introduction

Endosalpingiosis is a rare benign condition characterized by the ectopic proliferation of fallopian tube-like epithelium, most commonly in pelvic locations such as the uterus and ovaries. It is frequently associated with a history of prior pelvic surgery (1). The reported incidence varies depending on the diagnostic modality, ranging from 0.7% with histopathological diagnosis alone to 11% when magnetic resonance imaging (MRI) is utilized (2). Clinically, patients commonly present with chronic pelvic pain, often associated with cystic lesions or adhesions (3), and may exhibit concurrent ovarian cysts or reproductive tract abnormalities.

Diagnostic evaluation combines imaging and histopathology. Ultrasound typically demonstrates characteristic thin-walled anechoic cysts, while contrast-enhanced ultrasound (CEUS) provides a dynamic assessment of cyst wall vascularity (4). MRI further differentiates lesions through distinctive signal patterns, including T2-weighted hyperintensity and T1-weighted hypointensity (5, 6).

Due to diagnostic challenges and limited therapeutic awareness, current management primarily relies on surgical intervention, such as hysterectomy or salpingo-oophorectomy (7). Emerging evidence of strong hormone receptor expression suggests the potential for fertility-sparing hormonal therapies, particularly in reproductive-aged patients.

## Case presentation

A 47-year-old woman presented with chronic pelvic pain persisting for 2 years, complicated by the recent onset of irregular vaginal bleeding over the past 2 months.

### Medical history

The patient had a history of left tubal ectopic pregnancy treated with salpingostomy, left ovarian cystectomy for a benign ovarian cyst (specific type unknown), and one cesarean section. During both surgeries, no abnormal cystic masses were observed on the uterine surface. Her non-gynecologic history included hypertension managed with amlodipine. She was para 1 (vaginal delivery). No family history of gynecologic malignancies was reported.

### Gynecological examination

The external genitalia appeared normal, and the vagina was unobstructed with minimal odorless white discharge. The cervix was hypertrophic with multiple visible cysts. The uterus was retroverted with limited mobility. Bilateral adnexal regions were not palpable, and a non-tender mass was noted in the posterior fornix.

### Laboratory findings

Tumor markers were within normal ranges (CA125: 39.9 U/mL, HE4: 56.32 pmol/L, AFP: 1.1 ng/mL). The cervical cancer screening was negative.

### Imaging studies

**Figure 1:**
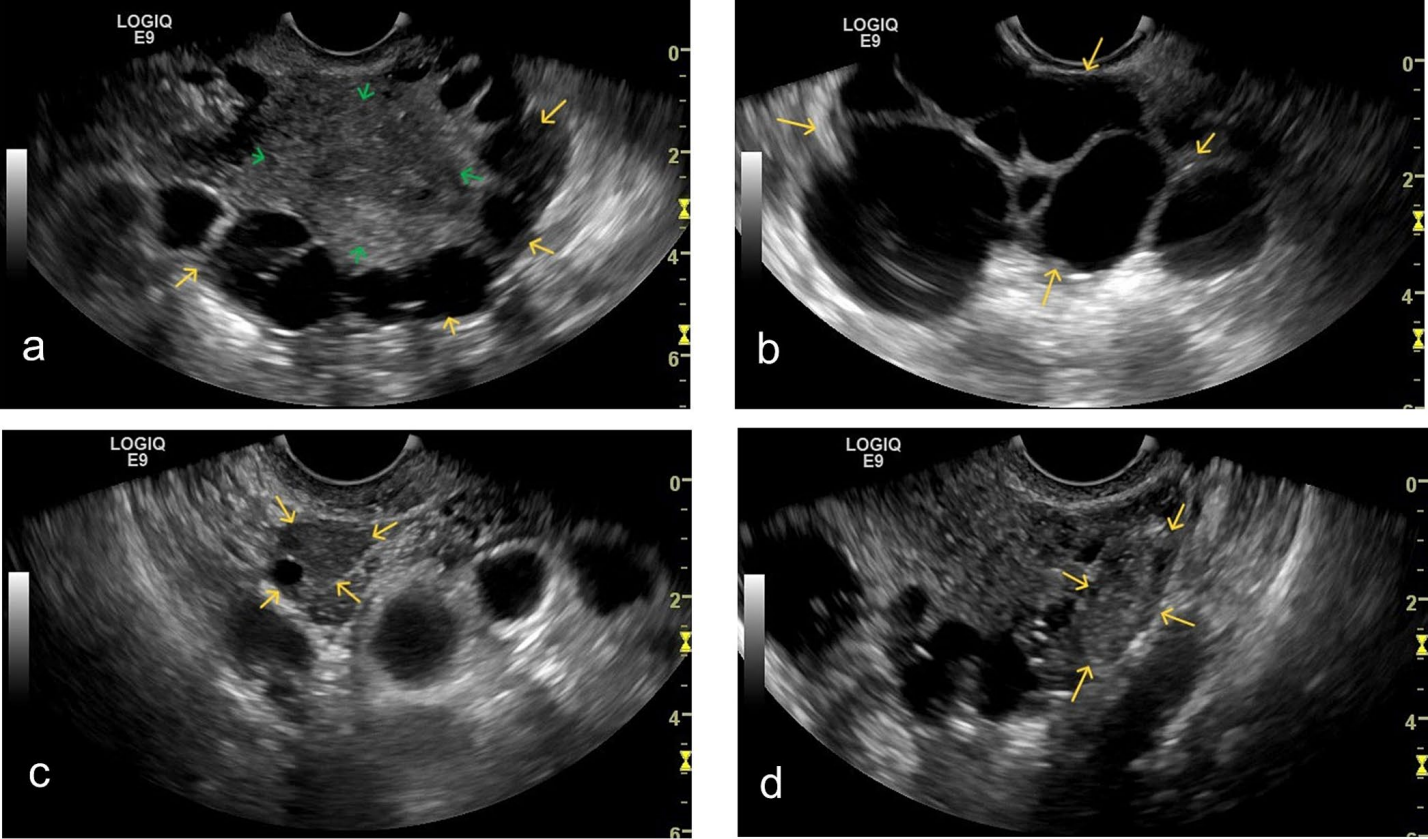
Grayscale ultrasound findings: **(a)** Grayscale ultrasound shows multiple cystic anechoic areas (yellow arrows) of varying sizes within the myometrium (green arrow), located near the subserosal region. The cystic areas exhibit homogeneous anechoic content. **(b)** Multiple cystic anechoic areas (yellow arrows) are observed in the parametrium. The cyst walls are thin and irregular, with homogeneous anechoic content inside. **(c)** The right ovary is visualized (yellow arrow). Upon gentle movement of the transvaginal probe, the right ovary demonstrates good relative mobility with the surrounding tissues. **(d)** The left ovary is visualized (yellow arrow). Upon gentle movement of the transvaginal probe, the left ovary appears adherent to the adjacent cystic structures.

B-mode ultrasound revealed multiple anechoic cystic areas within the myometrium (10–40 mm in diameter), located adjacent to the serosal surface without communication with the uterine cavity (Figure 1a). These oval-shaped lesions exhibited thin, smooth walls without papillary projections. The largest cross-section demonstrated more than 15 cystic areas. Additional anechoic cystic areas were observed near the uterus with tissue adhesions (Figure 1b). The endometrium measured 10 mm in thickness with heterogeneous echogenicity. Both ovaries were visualized; the right ovary showed mobility (Figure 1c), while the left ovary displayed adhesions (Figure 1d). No significant pelvic free fluid was identified.

**Figure 2:**
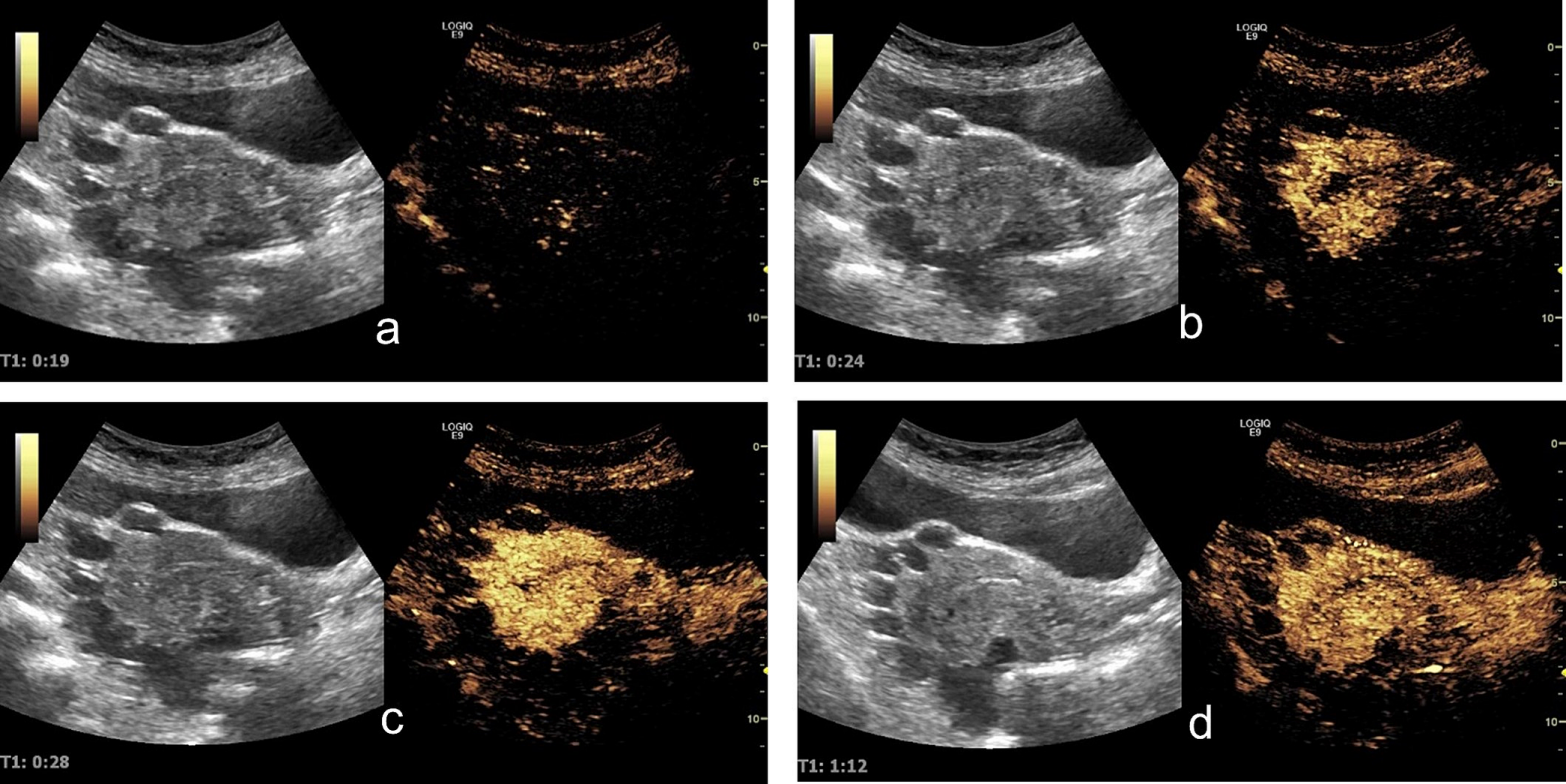
Contrast-enhanced ultrasound findings: **(a)** At 19 s, the uterine myometrium begins to enhance. **(b)** At 24 s, the uterine myometrium shows significant enhancement, while no obvious enhancement is observed in the lesion walls or the endometrium. **(c)** At 28 s, the lesion walls and endometrium begin to enhance, with the uterine myometrium showing marked enhancement. **(d)** At 72 s: The contrast agent demonstrated a washout phase in the lesion capsule wall, endometrium, and myometrium.

Contrast-enhanced ultrasound (CEUS) demonstrated sequential enhancement: myometrial enhancement began at 19 s (Figure 2a), with significant enhancement at 24 s (Figure 2b), followed by cyst wall and endometrial enhancement and marked myometrial enhancement (Figure 2c). The washout phase occurred at 72 s in the lesion capsule wall, endometrium, and myometrium (Figure 2d). The cysts displayed no internal enhancement throughout the examination.

**Figure 3:**
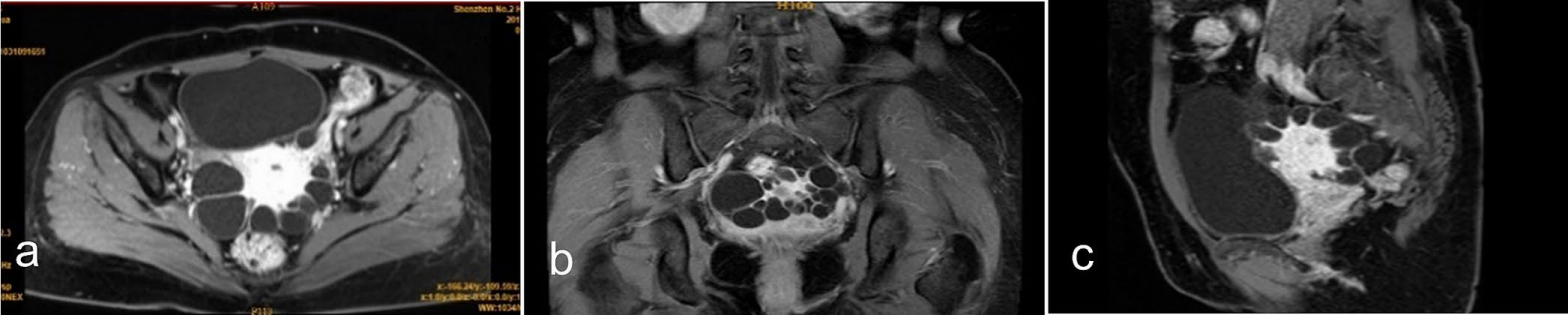
Contrast-enhanced MRI findings: **(a)** Axial view, **(b)** coronal view, and **(c)** sagittal view show the lesion located in the uterine myometrium near the serosal layer. The cyst wall exhibits mild enhancement compared to the uterine myometrium, while no enhancement is observed within the cystic content.

MRI revealed a normal-sized uterus with intact endometrium. The lesions varied in size and had no inner wall nodules. Post-contrast imaging showed mild cyst wall enhancement without internal enhancement. Some lesions appeared to connect to the serosal layer. Bilateral adnexal regions were poorly visualized (Figure 3). Imaging studies were inconclusive for a definitive diagnosis.

### Treatment

The patient underwent exploratory laparotomy, total hysterectomy, bilateral salpingo-oophorectomy, omentectomy, appendectomy, and pelvic adhesiolysis. Intraoperative exploration revealed multiple grayish-white cystic lesions (2–25 mm in diameter) on the uterine serosal surface, with thin-walled architecture and clear fluid content, adherent to surrounding tissues. No additional pathological findings were identified in the pelvic cavity.

### Pathological findings

Gross examination showed multiple gray-white vesicles (2–25 mm in diameter) on the uterine serosal surface. The cyst walls measured approximately 1 mm in thickness and contained clear fluid, with no obvious proliferative lesions on the inner walls.

**Figure 4:**
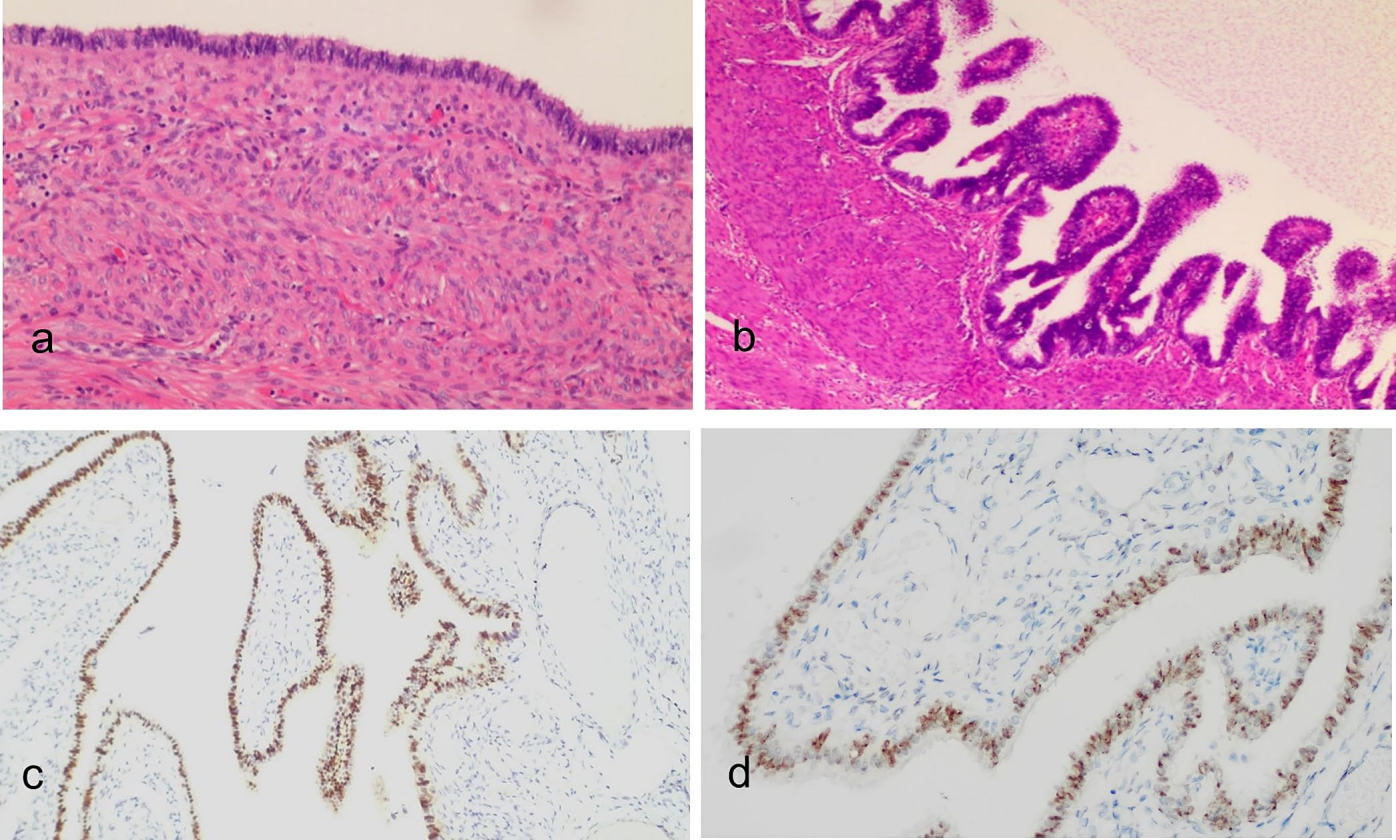
Histopathological images of the lesion (H&E staining): **(a)** Low-power view and **(b)** medium-power view show the cyst wall of the lesion in the uterine myometrium lined with tubal-like pseudostratified ciliated columnar epithelial cells. Immunohistochemical analysis revealed that the epithelial cells were positive for PAX-8 **(c)** and WT-1 **(d)**. Mucin was present in the apical cytoplasm and was retained within the glandular lumens.

Histological examination revealed multiple myometrial cysts lined with pseudostratified ciliated columnar epithelium (Figures 4a,b), consistent with endosalpingiosis cysts. Immunohistochemical analysis demonstrated positive expression of PAX-8 (Figure 4c), WT-1 (Figure 4d), ER (~95%), PR (~95%), Ki67 (~2%), P53 (weak, ~35%), and CK7, while CK20 and CDX2 were negative, supporting a tubal origin and hormone responsiveness.

### Follow-up

The patient was followed up for 6 years postoperatively. During this time, she reported no recurrence of pelvic pain or abnormal vaginal bleeding. Annual imaging studies, including ultrasound and MRI, revealed no recurrence of cystic lesions or other pelvic abnormalities. Her general health remained stable, and no additional gynecologic interventions were required. This follow-up supports the benign nature of florid cystic endosalpingiosis and emphasizes the importance of accurate diagnosis to avoid unnecessary radical interventions.

## Discussion

Endosalpingiosis, first described by Sampson in 1930 (8), is a rare benign condition characterized by the presence of fallopian tube-like epithelium in ectopic locations. This case presents unique features that contribute to the current understanding of the condition. The patient’s history of left tubal ectopic pregnancy treated with salpingostomy aligns with previously reported risk factors. However, unlike most reported cases where endosalpingiosis coexists with endometriosis and cervical endometriosis as part of the Müllerian system non-neoplastic lesion triad (9), this case demonstrated isolated myometrial involvement without evidence of other pelvic endometriotic lesions.

The symptoms associated with endosalpingiosis can vary, with chronic pelvic pain being the most common complaint (3). Consistent with the literature, this case presented with chronic pelvic pain, although approximately 30% of patients are asymptomatic (3). Radiologically, the MRI findings in this case, including high signal intensity on T2-weighted images, low signal intensity on T1-weighted images, and mild enhancement of cyst walls on contrast-enhanced sequences, are consistent with the features of endosalpingiosis described in previous studies (6). However, the dynamic enhancement pattern observed on contrast-enhanced ultrasound (CEUS)—characterized by delayed enhancement of cyst walls with lower peak intensity compared to the myometrium—represents a novel finding not previously reported.

The differential diagnosis of uterine myometrial cysts encompasses a variety of conditions, both congenital and acquired, requiring careful evaluation. For example, cystic adenomyosis typically exhibits thickened cyst walls with periodic bleeding, reflecting the presence of ectopic endometrial tissue within the myometrium. Cystic degeneration of uterine fibroids often shows irregular patterns and lacks the thin, smooth walls characteristic of endosalpingiosis. Furthermore, differentiation from metastatic tumors is essential, as metastatic lesions frequently display solid components visible on contrast-enhanced imaging. In contrast, endosalpingiosis is characterized by thin cyst walls and the absence of solid enhancement, which supports its benign nature. In this case, the thin cyst walls, smooth enhancement pattern, absence of solid components, and lack of periodic bleeding on imaging allowed differentiation from other possible diagnoses.

Histopathological examination, considered the gold standard for diagnosis (10), confirmed endosalpingiosis by identifying characteristic pseudostratified ciliated columnar epithelium lining the cyst walls. This case highlights the potential for underdiagnosis, as small pelvic cystic lesions may often be overlooked and not subjected to histological evaluation (11). The involvement of extrauterine sites, such as the visceral peritoneum, appendix, or pelvic lymph nodes, has been reported in the literature (12–14). However, in this case, there was no evidence of endosalpingiosis involving the bowel, bladder, or visceral peritoneum.

The biological nature of endosalpingiosis remains incompletely understood, although it is rare in children and postmenopausal women (15). Immunohistochemical findings in this case revealed strong positivity for both estrogen (~95%) and progesterone receptors (~95%), indicating hormone responsiveness. These findings support the hypothesis that, like endometriosis, endosalpingiosis may be hormone-dependent.

Due to limited awareness and diagnostic uncertainty, current management strategies for myometrial endosalpingiosis remain largely surgical. The majority of cases reported in the literature have involved extensive surgeries, including total hysterectomy, bilateral salpingo-oophorectomy, omentectomy, appendectomy, and pelvic lymphadenectomy (7). While our patient underwent extensive surgical treatment, the strong hormone receptor expression observed in this case suggests potential opportunities for hormone-based therapeutic approaches, especially in women of reproductive age.

According to the literature, most cases of myometrial endosalpingiosis exhibit benign biological behavior and predominantly occur in reproductive-age women (16, 17). This aligns with the clinical course of our patient, whose 6-year follow-up revealed no recurrence or related complications, further supporting the benign nature of endosalpingiosis. Enhanced clinical awareness of this condition is essential to avoid overtreatment and preserve fertility when appropriate. Establishing a multicenter research database would help better define the natural history, diagnostic criteria, and optimal management strategies of this condition. Future studies could explore more conservative treatment approaches tailored to younger women wishing to preserve fertility.

This study, however, has certain limitations. As a single-case report, the conclusions drawn are not generalizable and require validation in larger studies. Additionally, the lack of intraoperative imaging documentation limits the ability to verify the visual characteristics of the lesions observed during surgery. Future studies with larger sample sizes and well-documented imaging data are recommended to provide a deeper understanding of the condition and its management.

## Conclusion

This case report provides significant insights into myometrial endosalpingiosis. Our findings demonstrate that endosalpingiosis can occur independently without other Müllerian anomalies. The CEUS enhancement patterns offer a novel diagnostic approach to differentiating this condition from other cystic uterine lesions. Long-term follow-up results support the current consensus that this is a benign condition. Most importantly, increased awareness of this entity among clinicians is crucial to prevent misdiagnosis as malignancy and avoid unnecessary radical surgery, particularly in reproductive-age women where fertility preservation is essential. Further studies are needed to establish standardized diagnostic criteria and evaluate conservative management approaches for this rare but clinically significant condition.

## Data Availability

The original contributions presented in the study are included in the article/supplementary material, further inquiries can be directed to the corresponding author.

## References

[1] Peixinho C Machado-Neves R Silva PT Bernardes J Silva AC Amaro T. Hysteroscopic findings related with the assessment and treatment of uterine florid cystic endosalpingiosis: a case report and review of all the published cases. Acta Medica Port. (2021) 34:868–73. doi: 10.20344/amp.14292, PMID: 32991276

[2] Buck Louis GM Hediger ML Peterson CM Croughan M Sundaram R Stanford J. Incidence of endometriosis by study population and diagnostic method: the ENDO study. Fertil Steril. (2011) 96:360–5. doi: 10.1016/j.fertnstert.2011.05.087, PMID: 21719000 PMC3143230

[3] Singhania N Janakiraman N Coslett D Ahmad N. Endosalpingiosis in conjunction with ovarian serous cystadenoma mimicking metastatic ovarian malignancy. Am J Case Rep. (2014) 15:361–3. doi: 10.12659/AJCR.890921, PMID: 25180540 PMC4159242

[4] Stoelinga B Dooper AMC Juffermans LJM Postema AW Wijkstra H Brölmann HAM. Use of contrast-enhanced ultrasound in the assessment of uterine fibroids: a feasibility study. Ultrasound Med Biol. (2018) 44:1901–9. doi: 10.1016/j.ultrasmedbio.2018.03.030, PMID: 29735316

[5] Abuhamad A. Ultrasound in obstetrics and gynecology: a practical approach. 1st ed. Norfolk, VA: Society of Maternal-Fetal Medicine (2014).

[6] Calagna G Cucinella G Tonni G Gregorio RD Triolo O Martorana A. Cystic adenomyosis spreading into subserosal-peduncolated myoma: how to explain it? Int J Surg Case Rep. (2015) 8c:29–31. doi: 10.1016/j.ijscr.2015.01.005, PMID: 25617727 PMC4353969

[7] Im S Jung JH Choi HJ Kang CS. Intramural florid cystic endosalpingiosis of the uterus: a case report and review of the literature. Taiwan J Obstet Gynecol. (2015) 54:75–7. doi: 10.1016/j.tjog.2014.11.011, PMID: 25675925

[8] Sampson JA. Postsalpingectomy endometriosis (Endosalpingiosis). Am J Obstet Gynecol. (1930) 20:443–80. doi: 10.1016/S0002-9378(16)42561-5

[9] Gallan AJ Antic T. Benign müllerian glandular inclusions in men undergoing pelvic lymph node dissection. Hum Pathol. (2016) 57:136–9. doi: 10.1016/j.humpath.2016.07.003, PMID: 27438608

[10] Esselen KM Ng SK Hua Y White M Jimenez CA Welch WR. Endosalpingiosis as it relates to tubal, ovarian and serous neoplastic tissues: an immunohistochemical study of tubal and Müllerian antigens. Gynecol Oncol. (2014) 132:316–21. doi: 10.1016/j.ygyno.2013.12.007, PMID: 24333360

[11] Fukunaga M. Tumor-like cystic endosalpingiosis of the uterus with florid epithelial proliferation. A case report. Apmis. (2004) 112:45–8. doi: 10.1111/j.1600-0463.2004.apm1120108.x, PMID: 14961974

[12] Zinsser KR Wheeler JE. Endosalpingiosis in the omentum: a study of autopsy and surgical material. Am J Surg Pathol. (1982) 6:109–18. doi: 10.1097/00000478-198203000-00003, PMID: 7102891

[13] Cajigas A Axiotis CA. Endosalpingiosis of the vermiform appendix. Int J Gynecol Pathol. (1990) 9:291–5. doi: 10.1097/00004347-199007000-00009, PMID: 2373590

[14] Laufer MR Heerema AE Parsons KE Barbieri RL. Endosalpingiosis: clinical presentation and follow-up. Gynecol Obstet Investig. (1998) 46:195–8. doi: 10.1159/000010032, PMID: 9736803

[15] Schuldenfrei R Janovski NA. Disseminated endosalpingiosis associated with bilateral papillary serous cystadenocarcinoma of the ovaries. A case report. Am J Obstet Gynecol. (1962) 84:282–9. PMID: 13909448

[16] Morales-Roselló J Pamplona-Bueno L Montero-Balaguer B Desantes-Real D Perales-Marín A. Florid cystic endosalpingiosis (Müllerianosis) in pregnancy. Case Rep Obstet Gynecol. (2016) 2016:1–4. doi: 10.1155/2016/8621570, PMID: 27668111 PMC5030444

[17] Nixon KE Kenneth Schoolmeester J Bakkum-Gamez JN. Florid cystic endosalpingiosis with uterine preservation and successful assisted reproductive therapy. Gynecol Oncol Rep. (2018) 25:8–10. doi: 10.1016/j.gore.2018.05.003, PMID: 30014018 PMC6019862

